# Acute promyelocytic leukemia in children cancer hospital Egypt

**DOI:** 10.1007/s12672-024-01037-6

**Published:** 2024-06-11

**Authors:** Samah Semary, Mahmoud Hammad, Dina Yassin, Nahla El Sharkawy, Sonya Soliman, Sherine Salem, Emad Ezzat, Ahmed Mosa, Sonia Ahmed

**Affiliations:** 1 Department of Clinical Oncology, Faculty of Medicine, Beni-Suef University, Beni-Suef, Egypt; 2 Department of Pediatric Oncology, Children’s Cancer Hospital Egypt (CCHE-57357), Cairo, Egypt; 3 Department of Pediatric Oncology, National Cancer Institute (NCI), Cairo University, Cairo, Egypt; 4 Department of Clinical Pathology, National Cancer Institute (NCI), Cairo University, Cairo, Egypt; 5 Department of Clinical Pathology, Children’s Cancer Hospital Egypt (CCHE-57357), Cairo, Egypt; 6 Department of Clinical Pharmacy, Children’s Cancer Hospital Egypt (CCHE-57357), Cairo, Egypt; 7 Department of Clinical Research, Children’s Cancer Hospital Egypt (CCHE-57357), Cairo, Egypt; 8 Cairo, Egypt

**Keywords:** Acute myeloid leukemia, Acute promyelocytic leukemia, Translocation (15, 17), All-trans retinoic acid, Differentiation syndrome

## Abstract

**Background:**

Pediatric acute promyelocytic leukemia (APL) accounts for 5 to 15% of all myelocytic leukemia. A retrospective analysis of pediatric patients diagnosed and treated with APL was conducted at CCHE from July 2012 to the end of December 2019, to report the prevalence, clinical characteristics, results, and risk factors associated with induction failure and early death.

**Result:**

Sixty-two patients were reported, with an age greater than ten, an initial poor coagulation profile, and a total leukocyte count (TLC) greater than 30 10^3^/mm^3^ influencing 5-year overall (OS) and event-free survival (EFS), as well as a high promyelocyte count affecting 5-year EFS. Patients received a regimen based on the COG AAML0631 protocol. High-risk patients with an initial TLC > 10 × 10^3^/mm^3^ and an initial promyelocytic count of 30% or more with a substantial P-value are prognostic markers for early death during induction. In females, wild FLT3 increases the risk of differentiation syndrome (DS). Receiving steroids with all-trans retinoic acid (ATRA) induction may reduce the occurrence of DS. Relapse alters the outcome. In the current study, 45 patients are alive in complete remission, with a 5-year OS of 72.5% and a 5-year EFS of 69.4%, respectively.

**Conclusion:**

Pediatric APL outcomes are influenced by age above 10, an initial poor coagulation profile, and a promyelocyte count of more than 10%. An initial leukocyte count of more than 10 × 10^3^/mm and an initial promyelocytic count of more than 30% increase the risk of early death. Receiving steroids with ATRA may reduce the occurrence of DS.

**Supplementary Information:**

The online version contains supplementary material available at 10.1007/s12672-024-01037-6.

## Background

Pediatric Acute promyelocytic leukemia (APL) accounts for 5–15% of all myelocytic leukemia, having a low prevalence, better response to therapy, and survival outcome as compared to other acute myeloid leukemia (AML) subtypes [1–3]. The morphology corresponds to the M3 or M3v (hypo granular variant) subtype of the Franco-American-British (FAB) classification. Cytogenetically, translocation between chromosome 15 and 17 t (15; 17) (q22; q21) is common in about ninety-five percent of APL cases, and the fusion between the PML gene and the retinoic acid receptor alpha gene in chromosome 1, giving rise to the formation of the PML-RARa protein. This mutation blocks cell differentiation and causes more proliferation. The high incidence of deaths during induction chemotherapy needs further research to assess causes and prognostic factors. This study aims to report the prevalence, clinical features, outcomes, and risk factors causing induction failure and early deaths. Factors causing an increase in Differentiation Syndrome (DS) among patients with APL will be reported at Children Cancer Hospital Egypt from July 2012 till December 2019.

### Patients and methods

It is a retrospective study that reports on all pediatric patients under the age of 18 who were diagnosed and treated with acute promyelocytic leukemia (APL) at Children Cancer Hospital Egypt between July 2012 and the end of December 2019. The International Review Board has approved the project. Before beginning the chemotherapy protocol, the patients' guardians sign a declaration of permission.

The patient’s characteristics as age, gender, and obesity, laboratory data as initial peripheral blood total leukocyte count (TLC) which was above or below 10 × 10^3^/mm^3^ to classify the patient as standard or high risk, initial peripheral blood promyelocytic percent, the coagulation profile results, bone marrow aspirate (BMA) was performed for morphologies, immunophenotyping for detection of CD13, CD33, CD117, CD56, CD2, and cytogenetic for t(15;17) (q24.1;q21.1) and PML-RARa, RAR breakpoint (bcr1,bcr2,bcr3), the presence of FLT3/ITD mutation or wild type, and cerebrospinal fluid (CSF) cytology were collected.

Prognostic factors affecting and causing early induction deaths were assessed, including white blood cell count, promyelocyte percent in peripheral blood, initial platelet count, coagulopathies, RAR breakpoint subtype, FLT3-ITD mutation, the presence of differentiation syndrome (DS), its impact on patient outcome, and whether prophylactic steroids will reduce the incidence of DS.

All patients began the chemotherapy program based on the COG AAML0631 protocol, which used ATRA rather than Arsenic Trioxide, which is not available in our center [4].

All patients began treatment with induction, ATRA (25 mg/m^2^/day, Days 1–30, Per Oral (PO) twice a day (BID) had to be started once the presence of APL disease was suspected, IDA-rubicin (IDA): (12 mg/m^2^/dose) or (0.4 mg/kg/dose) IV Infusion/30 min, supplementary Fig. 1, and some patients received prophylactic dexamethasone to prevent DS.

If the white blood cell count (WBC) was initially greater than 10,000/mm^3^, hydroxyurea and dexamethasone were administered to decrease leukocytosis, disseminated intravascular coagulopathy (DIC), and reduce the likelihood of DS symptoms.

After induction, bone marrow was collected to determine morphological response, and all patients were treated with consolidation I (intrathecal (ITH) + ATRA + high dosage cytarabine (HD Ara-C) + mitoxantrone (MITOX)) and consolidation II (ITH + ATRA + IDA) [4], Supplementary Fig. 1.

The real-time quantitative reverse transcriptase polymerase chain reaction (RQ-PCR) of promyelocytic leukemia protein-retinoic acid receptor alpha (PML-RARα) testing is a tool for the diagnosis of PML-RARα fusion gene transcript, which allows for assessment of the disease response to treatment and detection of minimal residual disease (MRD), cytogenetic and molecular relapse. PCR was performed post-consolidation II in standard-risk patients; if negative, they will proceed to maintenance, and omitting consolidation III, but if positive, they will proceed to consolidation III, whereas all high-risk patients receive consolidation III (ITH + ATRA + IDA + HD Ara-C) and are evaluated by PCR post-consolidation III, Supplementary Fig. 1.

All patients proceed to maintenance 9 cycles of chemotherapy (each cycle lasts for 12 weeks—a total of 108 weeks), with ITH (ARA-C) given in cycle one only. ATRA: 25 mg/m^2^/day, PO split BID, 2 weeks daily (every 3 months), Mercaptopurine (6-MP) daily, and Methotrexate (MTX) weekly; bone marrow aspirate for RQ-PCR assay should be performed after each maintenance cycle (every 3 months) for all patients [4], Supplementary Fig. 1.

The end of treatment date was defined to calculate the time (early/late (pre or post-18 months from diagnosis)) of relapse if it occurred, the type of relapse (bone marrow (BM), central nervous system (CNS), or combined relapse), the line of treatment given post relapse, the response to chemotherapy cycles detected by RQ-PCR of PML-RARα testing, assess bone marrow transplantation (BMT) offered to relapsed patients, which type of BMT, autologous-BMT versus allogeneic-BMT, the state of life (in complete remission, relapsed, palliative, death), finally the statistical analysis of survival (5 years overall and event-free survival), and the analysis of all prognostic factors affecting the overall and event-free survival, factors causing early deaths, and factors increasing the incidence of differentiation syndrome.

### Definitions

- Risk stratification as standard risk: Patients with an initial WBC count of less than 10,000/µL are considered standard risk [5].
- Risk stratification as high risk: Patients with initial WBC greater than or equal to 10,000/µL detected by CBC at initial diagnosis are classified as high risk [5].
- Promyelocyte category: Acute promyelocytic leukemia (APL) is characterized by hypercellular bone marrow, with APL promyelocytes making up around 30% of myeloid cells in the classic variety [6].
- Early death: Death occurred within the first 30 days after diagnosis, before the end of the induction evaluation. [7].
- Complete remission (CR): The presence of blasts < 5%, less than 2% promyelocytes in bone marrow aspirate, no evidence of extra-medullary disease, transfusion independence, PCR negative, and a CBC showing absolute neutrophil count more than 1000/µL with platelet count more than 100,000/µL were all indicators of complete remission (CR) [8].
- Relapse disease: was identified by detecting more than 5% aberrant promyelocytes in the bone marrow aspirate, which typically exhibit numerous, irregular-appearing primary azurophilic granules [9].
- Relapse (early/late), pre or post-18 months apart from diagnosis [9].
- Differentiation syndrome (DS): This condition is associated with ATRA treatment and is characterized by unexplained fever, weight gain, respiratory distress, interstitial lung infiltrates, pleural and pericardial effusions, episodic hypotension, and acute renal failure [10].
- Unsatisfactory coagulation: A complex coagulopathy associated with acute promyelocytic leukemia, reflects consumptive coagulation as well as primary or secondary fibrinolysis which can cause intracranial or pulmonary hemorrhage [11].

In the current study, for all patients with a suspected diagnosis of APL at the initial workup, a coagulation profile was done to assess the low platelet count, prolonged prothrombin time (PT), affecting the partial thromboplastin time (PTT), low prothrombin concentration (PC), and high international normalized ratio (INR) to diagnose the bleeding disorder, which commonly present with APL, and may be affected by the presence of the disease, leading to an increased incidence of bleeding.

Body mass index: The Centers for Disease Control and Prevention (CDC) defined body mass index (BMI) as a person's weight in kilograms divided by their height in meters squared.

There are several techniques for calculating body mass index; at our institution, the BMI-for-age percentile is based on CDC growth charts for children and teenagers aged 2 to 19 years. The calculator takes the child’s sex, age, height, and weight as inputs.

### Statistical analysis

Overall survival (OS): Calculated from the date of first diagnosis to the date of death out of any cause or lost follow-up.

Event-free survival: Calculated from the date of initial diagnosis to the date of diagnosis of relapse or death.

The Kaplan–Meier method was used for survival analysis with 95% confidence intervals and values reported from the two-sided log-rank test.

Chi-squared and Fisher’s exact tests were used to evaluate the association between categorical variables.

The alpha error threshold (P-value) was set at 0.05. All statistical analyses were performed using IBM SPSS version 20.

## Results

From July 2012 to December 2019, 62 individuals under the age of 18 were diagnosed with APL out of 823 patients with AML (7.5%) at Children Cancer Hospital Egypt. The mean age was 9.2, with a median age of 9.9 and a range of 1.4 to 17.8, as shown in Supplementary Table 1.

Thirty-one (50%) of the patients were older than ten when they were diagnosed. The remaining patients were under the age of ten, with 5-year (5y) overall survival (OS) of 58.1% and 87.1%, respectively, with significant P-values of 0.01, Table 1, Supplementary Fig. 2, and 5y event-free survival (EFS) of 58.1% and 80.6%, with significant P-values of 0.043, Table 2, Supplementary Fig. 3.

**Table 1:** Patient characteristics (univariate analysis)

Initial variable	Count	Number of events(Death)	Overall survival (5 years) (%)	P-value with OS
Age group	62			**0.01**
Less than 10	31	4	87.1	
More than 10	31	13	58.1	
Obesity	59			0.5
Obese	15	5	66.2	
Not obese	44	10	77.2	
Steroid in induction	61			**0.058**
Yes	25	3	88.0	
No	36	13	63.9	
Hydroxyurea	59			0
Yes	10	7	30	
No	49	10	78.6	
Gender	62			**0.067**
Female	25	10	60.0	
Male	37	7	81.1	
BMA cellularity	61			0.8
Hypo-cellular	1	0		
Hyper-cellular	36	10	71.4	
Normo-cellular	24	6	74.4	
SR/HR				0.09
SR	35	7	80	
HR	27	10	63	
TLC (category) < or > 30 10^3^/mm^3^				**0.019**
No	44	9	79.5	
Yes	18	8	55.6	
Platlets_category < or > 50 × 10^3^/mm^3^				0.6
High	5	1	80	
Low	57	16	71	
Peripheral promyelocytes%				0.12
< 30%	33	6	80.9	
> 30%	29	10	65.5	
Promyelocytes % in peripheral blood (< or > 10%)	61			**0.059**
High	42	14	66.7	
Low	19	2	89.5	
Coagulation profile	62			**0.007**
Satisfactory	20	1	95	
Unsatisfactory	42	16	61.9	
PML RARa transcript subtypes				0.52
bcr3	**23**	**6**	**71.7**	
bcr2	**7**	**1**	**85.7**	
bcr1	**21**	**4**	**81**	
Not specific	**9**	**4**	**55.6**	
FLT3-ITD				0.3
Positive	**15**	**6**	**60**	
Negative	**40**	**9**	**75.8**	
Not done	**7**	**2**	**71.4**	
CD56				0.3
Positive	**7**	**1**	**85.7**	
Negative	**55**	**16**	**69.9**	
CD2				0.3
Positive	**11**	**4**	**63.7**	
Negative	**51**	**13**	**73.6**	
Differentiation syndrome				**0.9**
Affected	**31**	**9**	**71.0**	
Not affected	**31**	**8**	**74.2**	
Relapse				**0.07**
Yes	**6**	**4**	**33.3**	
No	**56**	**13**	**76.7**	
Types of second line protocol				
FLAG/ATRA	**2**	**2**		
FALG/M/ATRA	**1**	**1**		
FLAG-M	**1**	**0**		
Molecular PCR in relapse				
Positive	**3**			
Early relapse				
Yes	**1**	**1**	**0**	**0.025**
No	**5**	**3**	**33**	
Bone marrow transplant				0.45
Yes	**4**	**2**	**50**	
No	**58**	**15**	**74**	

Bold underlined values was for significant value

**Table 2:** Initial variables affecting event-free survival (univariate analysis)

	Events		PP-value E	5 years event-free survival (EFS) TION (%)
	0	1		
Age group				
< 10	25	6	**0.043**	80.6
> 10	18	13		58.10
Obesity				
Not obese	32	12	0.6	71.80
Obese	10	5		66.70
Steroid				
No	8	8		48
Yes	34	11	0.12	74.90
Gender				
Female	15	10		59
Male	28	9	0.13	74.50
Bone marrow cellularity				
Hyper cellular	25	11		68
Normo cellular	18	6	0.4	74.40
SR/HR				
HR	16	11		59.3
SR	27	8	**0.09**	77.1
TLC Less 30 10^3^ mm^3^				
No	34	10		75.70
Yes	9	9	0.12	94.40
Platelets category				
High(> 50 × 10^3^/mm^3^)	4	1		80
Low (< 50 × 10^3^/mm^3^)	39	18	0.6	67.10
Promyelocyte less 30%				
< 30%	25	7		77
> 30%	18	11	0.2	61.70
Promyelocytes category				
High	26	16	**0.035**	61.9
Low	17	2		89.5
Coagulation profile				
Unsatisfactory	24	18	**0.004**	57.1
Satisfactory	19	1		95
PML RARa transcript subtype				
bcr1	16	5		74.10
bcr2	6	1		85.70
Bcr3	16	7	0.63	67.30
Not Spec	5	4		55.60
FLT3Result				
Negative	29	11		70.20
Positive	9	6		60
Not done	5	2	0.4	71.40
Differentiation syndrome				
No	22	9		70.40
Yes	21	10	0.9	65.60

Bold underlined values was for significant value

Thirty-seven (59.7%) patients were males, with a male-to-female ratio of 1.48:1, and the 5-year OS for males and females was 81.1% and 60.0%, respectively, with a P-value of 0.067 (Table 1, Supplementary Fig. 4). The body mass index was measured to investigate the effect of obesity on patient outcomes; 15 patients were obese with no significant P-value, 0.5, Table 1.

The initial peripheral blood total leukocyte count (TLC) was evaluated to classify the patient as standard or high risk. 35 (56.4%) patients had an initial TLC < 10 × 10^3^/mm^3^ and were considered standard-risk patients, while 27 (43.5%) patients had an initial TLC > 10 × 10^3^/mm^3^ and were considered high-risk patients, with 5y OS 80% and 63% respectively with P-value 0.09 (Table 1, Supplementary Fig. 5), However, patients with an initial TLC greater than 30 × 10^3^/mm^3^ had a 5-year OS of 55.6%, with a significant P-value of 0.019 (Table 1, Supplementary Fig. 6). Patients with a high level of promyelocytes > 10%, or < 10% in the peripheral blood count had a 5y OS of 66.7%, 88.9% with a P-value of 0.059 (Table 1, Supplementary Fig. 7) and 5y EFS of 61.9%, and 89.5% respectively with a significant P-value of 0.035 (Table 2, Supplementary Fig. 8).

Initially, all patients underwent a coagulation profile. It was only satisfactory for 32% of patients. The remaining patients (67.7%) had unsatisfactory coagulation profiles affecting the bleeding tendency and DIC at the time of diagnosis, with 5 years OS 95%, 61.9%, with significant P-value, 0.007, Table 1, Supplementary Fig. 9, and 5 years EFS 95%, 57.1%, with significant P-value, 0.004, Table 2, Supplementary Fig. 10.

All patients had bone marrow aspiration for morphology and immunophenotyping for the presence of CD13, CD33, and CD117 for diagnosis. CD56 was identified in 7 (11%) individuals, with no significant P-value of 0.3 (Table 1). CD2 was positive in only 11 (17.7%) individuals, with no significant P-value of 0.3 (Table 1).

Cytogenetic analysis was used to discover translocations (15; 17) (q24.1; q21.1) and PML-RARα for correct diagnosis. RT-PCR was used to map RAR breakpoints (bcr1, bcr2, bcr3). Bcr3 was diagnosed in 23 (37%) patients, whereas bcr1 was detected in 21 (33.8%), with no significant P-value of 0.52, as shown in Table 1. The FLT3/ITD mutation was detected in 15 (24%) individuals, with no significant P-value (0.3) for survival result. Table 1.

Patients started on chemotherapy protocol adopted from COG AAML0631 chemotherapy protocol for APL using ATRA without using Arsenic Trioxide, which is not available in our center [4].

Except for one patient who died before treatment, all patients began induction treatment; ATRA was initiated once APL disease was suspected; the value of initially administering steroids with ATRA to reduce the incidence of DS was calculated as approximately 25 (40%) patients received steroids with ATRA, the 5y overall survival according to giving steroid with ATRA or not were, 88.0%, and 63.9% respectively with a P-value of 0.058, Table 1, supplementary Fig. 11.

Differentiation syndrome was detected throughout treatment in 31 (50%) patients, which affected the continuation of giving ATRA, followed by omitting or modifying the ATRA dose. The 5-year OS for patients who did not suffer from differentiation syndrome was 74.2%, and for patients who complained of recurrent differentiation syndrome was 71.0%, with no significant P-value (0.9, Table 1, Supplementary Fig. 12).

DS's diagnostic criteria were variable, including fever, respiratory distress, weight gain, edema, pleural or pericardial effusions, and episodic hypotension.

The diagnosis of DS is based on clinical symptoms; 44% of patients with DS had dyspnea and tachypnea, with or without fever. CT chest scans were performed, and some patients had pulmonary infiltrations with or without unilateral or bilateral pleural effusion. 54% of patients experienced neurotoxicity symptoms such as headaches, photophobia, blurred vision, or convulsions. CT brain revealed a pseudo-tumor cerebri, while fundus examination revealed papilledema.

Sixty-one patients received induction ATRA-IDArubicin, 50 (80%) patients received consolidation I, while 11 (17.7%) patients died during the first 30 days of induction and were considered as early death [6], Supplementary Table 2.

Out of the 11 patients who died during induction, 8 died as a result of intracranial hemorrhage, one died of vaginal hemorrhage, which was considered disease-related mortality, and the remaining two suffered from DS and died of respiratory distress, which was considered treatment-related mortality. All patients who died during induction died before day ten. Prognostic factors causing early deaths during induction were stated in Table 3 as follows: obesity did not affect early deaths in patients with APL in pediatric age as more deaths accrued in non-obese with a P-value of 0.02, Table 3, but high-risk patients with initial TLC > 10 × 10^3^/mm^3^ suffered from early deaths than standard risk with a significant P-value of 0.01, initial promyelocytic count above 30% affecting the outcome of the APL patients and causing early death with significant P-value, 0.018, Table 3. Gender, low initial platelet count, unsatisfactory coagulation profile, presence of FLT3-ITD mutation, and initiating steroid with ATRA to reduce the incidence of DS were not considered poor prognostic factors for causing early death, with no significant P-value, Table 3.

**Table 3:** Multivariate analysis for the prognostic factors causing induction early deaths

		Early death		P-value
		Obesity		
		Not Obese	Obese	
Early death	No	1	3	**0.02**
	Yes	9	2	
		SR/HR		
		HR	SR	
Early death	No	1	5	**0.01**
	Yes	9	2	
		Promyelocyte		
		< 30%	> 30%	
Early death	No	5	1	**0.018**
	Yes	2	9	
		Platelets category		
		High (> 50 × 10^3^/mm^3^)	Low (< 50 × 10^3^/mm^3^)	
Early death	No	1	5	0.35
	Yes	0	11	
		Coagulation profile		
		unsatisfactory	Satisfactory	
Early death	No	5	1	0.35
	Yes	11	0	
		Steroid in induction with ATRA		
		NO	YES	
Early death	No	5	1	0.69
	Yes	8	2	
		FLT3-ITD Results		
		Wild	Mutated	
Early death	No	5	1	0.1
	Yes	4	5	
		Gender		
		Female	Male	
Early death	No	4	2	0.5
	Yes	6	5	

Consolidation II was given to 50 (80.6%) patients (standard and high-risk patients); Supplementary Table 2, RQ-PCR of PML-RARα testing was done for 38 (76%) patients and was positive in 5 (13%) patients, with no significant P-value for death or relapse (0.38 and 0.47, respectively), Supplementary Table 3,4.

Nineteen (30.6%) patients got consolidation III (all high-risk patients and standard-risk patients with positive RQ-PCR after consolidation II); RQ-PCR was performed on 8 (42.1%) patients and was positive in 2 (3%), Supplementary Table 2.

The DS is the primary side effect of the chemotherapy protocol that impacts treatment continuation and, ultimately, disease outcome. The prognostic factors causing recurrent differentiation syndrome through treatment were reported, including an increased incidence of DS in females with a significant P-value of 0.01, Table 4, and in patients with wild FLT3 with a significant P-value of 0.04, Table 5, receiving steroids with ATRA may decrease the incidence of DS with a P-value of 0.058, Table 4.

**Table 4:** Multivariate analysis for the initial variables (age, gender, obesity, giving steroids with ATRA, and giving hydroxyurea initially) with differentiation syndrome

	Age group		P-value
	< 10	> 10	
Differentiation syndrome			
No	14	17	0.3
Yes	17	14	

	Obesity		P-value
	Not Obese	Obese	
Differentiation syndrome			
No	22	8	0.53
Yes	22	7	

	Steroid		P-value
	No	Yes	
Differentiation syndrome			
No	7	24	0.058
Yes	9	21	

	Hydroxyurea		P-value
	No	Yes	
Differentiation syndrome			
No	24	7	0.19
Yes	25	3	

	Gender		P-value
	Female	Male	
Differentiation syndrome			
No	6	25	0.01
Yes	19	12

**Table 5:** Multivariate analysis for the initial variables (promyelocyte counts, initial coagulation profile, and FLT3-ITD mutation) with differentiation syndrome

Initial variables with differentiation syndrome	Promyelocytes less than 30%		P-value
	< 30%	> 30%	
Differentiation syndrome			
No	14	17	0.3
Yes	18	12	

	Promyelocytes category		P-value
	High	Low	
Differentiation syndrome			
No	24	7	0.16
Yes	18	12	

	Initial coagulation profile		P-value
	Unsatisfactory	Satisfactory	
Differentiation syndrome			
No	19	12	0.2
Yes	23	8	

	FLT3-ITD result			P-value
	Wild	Mutated	Not done	
Differentiation syndrome				
No	16	9	6	0.04
Yes	24	6	1	

On the other hand, age, obesity, initial promyelocytic count, receiving hydroxyurea, and risk stratification did not increase the incidence of DS, with no significant P-value, Tables 4, 5, 6.

**Table 6:** Multivariate analysis for the initial variables (PML RARA transcript subtypes, and patient’s risk) with differentiation syndrome

	PML-RARa transcript subtypes				P-value
	bcr1	bcr2	bcr3	Not Spec	
Differentiation syndrome					
No	12	2	11	5	0.5
Yes	9	5	12	4	

	SR/HR		P-value
	HR	SR	
Differentiation syndrome			
No	14	17	1
Yes	13	18	

Six (9.7%) patients relapsed throughout the research period, with a 5-year OS of 33.3% (P-value of 0.07, Table 1, Supplementary Fig. 13). One patient experienced an early relapse, whereas the remainder had late relapses with a significant P-value of 0.025 (Table 1, Supplementary Fig. 14).

Relapsed individuals were treated with FLAG + -M + ATRA (fludarabine, high-dose cytarabine, with or without mitoxantrone). Bone marrow transplantation (BMT) was performed on four relapsing patients, autologous BMT was done for 2 patients, and allogeneic BMT for the other 2 patients, with 5y OS 50%, with no significant P-value, 0.45, Table 1.

Five-year event-free Survival (EFS) is not affected by obesity, gender, initial bone marrow cellularity, initial TLC, initial platelet count, risk stratification to standard or high risk, PML RARA transcript subtype, FLT3-ITD mutation, receiving steroid with ATRA, differentiation syndrome, with no significant P-value, Table 3.

In the current analysis, forty-five patients are surviving in complete remission, with a 5-year overall survival of 72.5% and a 5-year event-free survival of 69.4% (Supplementary Table 2).

## Discussion

Pediatric acute promyelocytic leukemia (APL) represents 5%—15% of all myelocytic leukemia and has a lower prevalence, better response to therapy, and survival rate than other acute myeloid leukemia (AML) subtypes [1–3].

From July 2012 to the end of December 2019, 62 pediatric patients under the age of 18 with APL were evaluated and treated at Children Cancer Hospital Egypt, out of 823 AML patients (7.5%).

In FG Andrade 2021, 163 (17.5%) patients with APL were investigated among 931 AML cases [12]. The median age of pediatric APL cases ranges from 9 to 12 years [13, 14], which agrees with our finding that the median age was 9.9 years old. Thirty-one (50%) patients were older than 10 years. The rest were less than 10 years at diagnosis, Patients over 10 years old may present with higher white blood cell (WBC) counts, which can indicate a more advanced stage of APL, resulting in a poorer overall and event-free survival than those under 10 years old, as agreed with Testi et al. [14].

Females are more prevalent in pediatric age than in adult age [15, 16]; in the current study, the male-to-female ratio is 1.48:1, with no statistical significance.

Obesity was found to have a significant impact on APL patients’ outcomes, with 81% being obese compared to 41.7% in the non-APL/AML group (P < 0.001). High body mass index (BMI) > 30 was found in 57% of APL patients compared to 31% in the non-APL/AML group (P = 0.01). However, neither obesity nor chemotherapy estimated by dose based on optimal body weight influenced the survival outcome [17], this was consistent with the current study since 15 (24%) individuals were obese with no significant P-value. Obesity is more prevalent in pediatric patients with APL than in the general population. Obesity impacts the outcomes in patients who received AAML1331 treatment. These findings emphasize the necessity for additional studies into the potential role of obesity in pediatric APL [18].

The initial peripheral blood total leukocytic count (TLC) was evaluated to identify the patient as standard or high risk, with no statistical significance; however, patients with an initial TLC more than 30 × 10^3^/mm^3^ had a lower 5-year OS with a significant P value. Sanz MA et al. demonstrated that the presence of white blood cells of more than 5000/mm^3^ or 10,000/mm^3^ at the outset is one of the most important prognostic variables in patients with APL and causes a high incidence of relapse [19]. In the current study, the presence of more than 10% promyelocytes had a significant effect on 5-year EFS.

Initially, all patients underwent a coagulation profile. It was unsatisfactory in (67.7%), affecting the bleeding propensity, disseminated intravascular coagulopathy (DIC) at the time of diagnosis, and impacting the 5 years OS and EFS with significant P- value.

The presence of altered expression of several coagulation factors in APL blasts may be the primary cause of coagulopathy upon diagnosis. The blasts show enhanced expression of tissue factors such as tissue plasminogen activator, cysteine protease, Annexin 2, and urokinase-like plasminogen activator receptor, resulting in hypercoagulability and increased fibrinolysis [20, 21].

The treatment of the underlying disease is the cornerstone of DIC management, as evidenced by the significance of early ATRA delivery in APL. Other aspects of management concentrate on supportive care aimed at lowering the risk of bleeding from blood product transfusions [22].

All patients underwent bone marrow aspiration for morphology and immunophenotyping diagnosis; CD56 is a unique marker found in 10% of APL cases and is associated with a poor prognosis, with a higher risk of relapse in adult patients [23]; in the current study, CD56 was detected in only 7 (11%) patients, with no significant P-value.

CD2 identification in APL was strongly linked with high leukocytosis (P = 0.004), shorter time to relapse and high relapse rate (P = 0.03), and lower overall survival (P = 0.07) than in patients with APL with negative CD2 detection [24]. In the current study, CD2 was positive in only 11 (17.7%) patients, with no significant P-value, which can be attributed to the small number of patients in each category.

A bone marrow aspirate was performed for cytogenetic detection of translocation (15; 17) (q24.1; q21.1) and PML-RARα for accurate diagnosis. RAR breakpoints (bcr1, bcr2, bcr3) were mapped by RT-PCR. Bcr3 was diagnosed in 23 (37%) patients, while bcr1 was detected in 21 (33.8%), with no significant P-value.

The long bcr-1, variation bcr-2, and short bcr-3 are all mentioned in Breen K.A. (2012). Bcr-1 and Bcr-3 are more common in APL patients, accounting for around 95%, while bCR-2 is seen in only 5% of APL patients [20]. There was no correlation between the different PML-RARA isoforms and the other clinical and laboratory features [25].

The identification of FLT3/ITD mutation in pediatric AML is often associated with a poor prognosis [26], with high initial WBC counts and a worse prognosis than those who had AML without the FLT3-ITD mutation [27]. In the current study, FLT3/ITD mutation was found in 15 (24%) of pediatric APL patients, with no significant P-value. According to Kutny et al. FLT3 mutations account for 43% of pediatric APL cases. The COG AAML0631 investigation found no link between FLT3 mutations and bleeding/clotting events that led to early induction death. However, FLT3 mutant patients had a considerably greater recurrence rate after Arsenic Trioxide (ATO) consolidation [28].

Patients began on a chemotherapy protocol based on the COG AAML0631 chemotherapy protocol for APL, which used chemotherapy with ATRA without Arsenic Trioxide, which is not available in our country with a 5-year overall survival of 72.5% and a 5-year event-free survival of 69.4%. In the Children’s Oncology Group AAML0631 trial, the overall 3-year survival rate was 94%, and with an event-free survival rate of 91%. For SR and HR patients with APL, overall survival was 98% versus 86% (P = 0.003), and EFS was 95% versus 83% (P = 0.03) [4]. In our center, the high number of early deaths (17.7%) during induction, high rate of recurrence (9.6%), and insufficient and inadequate supportive services in our low-middle income country may explain the variance in outcome.

Our country has two main centers that address pediatric oncology: The Children's Cancer Hospital (CCHE) Egypt and the National Cancer Institute (NCI) Egypt. Both follow the same procedure as COG AAML 0631 but without the use of Arsenic Trioxide, which is not available in this nation. However, there was no data on the outcomes of pediatric patients with APL treated in NCI, as the only published data for NCI is for adult patients with APL treated between 2007 and 2011 [29].

In the COG AAML 1331 trial, all patients received ATRA and arsenic trioxide during the induction and four consolidation rounds, but no maintenance medication was given. Only patients with high-risk APL were given four doses of idarubicin during induction. Patients at standard risk had a 2-year EFS of 98.0% and an OS of 99.0%. Patients at high risk had a 2-year EFS rate of 96.4% and an OS rate of 100% [30].

ATRA started once suspicion of APL diagnosis, the most common manifestations of DS are unexplained fever, dyspnea, water retention resulting in weight gain of more than 5 kg, laboratory findings showing elevated WBC, acute renal insufficiency, and radiological diagnosis as pleural or pericardial effusion detected by chest x-ray [31].

The most prevalent symptoms used to diagnose DS in the current study were fever, dyspnea, tachypnea, and neurotoxicity symptoms such as headache, photophobia, blurred vision, or convulsion. A CT chest scan confirms the diagnosis of DS when pulmonary infiltrations or pleural effusion are present. CT brain revealed a pseudo-tumor cerebri, while fundus examination revealed papilledema.

Early detection, examination, and therapy with corticosteroids can save lives in the management of the life-threatening illness DS [31]. In the current study, 21 (46.6%) of 45 patients who received prophylactic steroids initially developed a DS. In comparison, 9 (56.2%) of 16 patients who did not receive prophylactic steroids had DS; when comparing the two groups, prophylactic steroids may reduce DS with a P-value of 0.058.

Differentiation syndrome was discovered multiple times throughout therapy, affecting the continuation of ATRA administration, followed by omission or change of the ATRA dose, which may have an impact on the APL patient's result. The 5-year OS for patients who did not have DS was 74.2%, and for those who complained of recurrent DS was 71.0%, with no significant P-value; this finding was also mentioned by Sultana et al. [31]. On the other hand, age, obesity, starting promyelocytic count, hydroxyurea treatment, and risk stratification did not increase the prevalence of DS.

Alessandro Molinaro et al. conducted an investigation that revealed that WBC counts more than 5 × 10(9)/L and a higher serum creatinine level were associated with an elevated risk of severe DS. Still, patients who use steroids as prophylactic had a lower incidence of severe and recurring DS [32]. Severe DS had a worse 7-year relapse-free survival rate in the LPA96 study (60% vs 85%, P = 0.003), [33].

Despite treatment advancements and appropriate supportive care, early death (ED) during induction remains the most significant factor influencing APL survival [34]. Male gender (P = 0.01), initial white leukocyte counts > 10 × 109/L (P = 0.03), fibrinogen level < 1.5 g/L (P = 0.02), and delayed ATRA administration more than 24 h after diagnosis and hospital admission (P < 0.001) were all substantially linked with ED [34].

In the current study, ED occurred in 17.7% of the study patients, and the prognostic factors causing early deaths during induction were evaluated; obesity did not affect early deaths in pediatric patients with APL with P-value 0.02, but high-risk patients with initial TLC > 10 × 10^3^/mm^3^ suffered from earlier deaths than the standard risk with a significant P-value 0.01, and initial promyelocytic count above 30% affected the outcome of the APL patients and causing early death with a significant P-value, of 0.018, these findings are with Testa’s and Molinaro [23].

Gender, low initial platelet count, suboptimal coagulation profile, FLT3-ITD mutation, and beginning steroid with ATRA to reduce the incidence of DS are not considered bad prognostic indicators for early death.

In our low-middle-income country, insufficient and inadequate supportive facilities, as well as a scarcity of beds in intensive care units, may contribute to the high prevalence of induction early mortality.

Treatment of APL in pediatric patients with ATRA in combination with chemotherapy results in full response rates of more than 90% and long-term overall survival of more than 80%. Testi M et al. stratified patients into standard-risk (SR) and high-risk (HR) groups based on initial WBC counts and used an ATRA-dependent protocol. Without ATO, the cumulative dose of anthracyclines in SR and HR patients was lower than in other studies (355 mg/m^2^ and 405 mg/m^2^, respectively). The five-year overall and event-free survival rates for the entire group were 94.6% and 79.9%, respectively. These findings show that prolonged treatment of ATRA, along with restricted anthracycline exposure, results in significant cure rates in childhood APL [3]. In the current study, 45 patients are in complete remission, with a 5-year overall survival rate of 72.5% and a 5-year event-free survival rate of 69.4%. This is characterized by a high rate of induction mortality (17.7%), a high incidence of relapse (9.6%), and a lack of supportive care.

## Conclusion

A retrospective analysis of pediatric patients diagnosed and treated with acute promyelocytic leukemia (APL) at Children Cancer Hospital Egypt from July 2012 till the end of December 2019. Patients began following a treatment based on the COG AAML0631 chemotherapy protocol. The outcome of pediatric APL is influenced by age above 10, an initial poor coagulation profile, and a promyelocyte level of more than 10%. Prognostic factors causing early deaths during induction of patients categorized as high-risk patients with an initial total leukocyte count of 10 10^3^/mm^3^ and an initial promyelocytic count of more than 30% with a significant P-value.

Female gender and wild FLT3 increase the incidence of differentiation syndrome, with significant P-values. Receiving steroids alongside ATRA may reduce the prevalence of ATRA syndrome.

Relapse influences the outcome. Bone marrow transplantation was performed on four of the relapsed patients, with a 5-year OS of 37% and no significant P-value. In the current study, 45/62 patients are alive in complete remission, with a 5-year OS of 72.5% and a 5-year EFS of 69.4%, respectively, due to a high frequency of induction mortality of 17.7%, a high relapse rate of 9.6%, and insufficient and inadequate supportive services in our low-middle income country.

### Supplementary Information

Additional file 1 (DOCX 216 KB)

## Data Availability

All authors agree to make the raw data and materials described in our manuscript freely available to any scientist wishing to use them for non-commercial purposes, as long as this does not breach participant confidentiality.

## Abbreviations

APL: Acute promyelocytic leukemia
TLC: Total leukocyte count
OS: Overall Survival
EFS: Event-free survival
COG: Children’s Cancer Group
AML: Acute myeloid leukemia
ATRA: All trans-retinoic acid
FAB: Franco-American-British
T: Translocation
PML-RARa: Promyelocytic leukemia protein-retinoic acid receptor alpha
FLT3/ITD: FLT3/Internal tandem duplication
CSF: Cerebro-spinal fluid
PO: Per oral
BID: Twice a day
WBC: White blood cells
IDA: IDArubicin
ITH: Intrathecal
HD: High dose
Ara-C: Cytarabine
MITOX: Mitoxantrone
RQ-PCR: Real-time quantitative reverse transcriptase polymerase chain reaction
PCR: Reverse transcriptase polymerase chain reaction
MRD: Minimal residual disease
6-MP: Mercaptopurine
MTX: Methotrexate
BMA: Bone marrow aspirate
CNS: Central nervous system
BMT: Bone marrow transplantation
Auto-BMT: Autogenic bone marrow transplantation
Allo-BMT: Allogenic bone marrow transplantation
CBC: Complete blood count
CR: Complete remission
BMI: Body mass index
CDC: Centers for Disease Control and Prevention
IBM SPSS: International Business Machines-Statistical Package for the Social Sciences
FLAG-ATRA: Fludarabine, high dose cytarabine with all trans retinoic acid
DIC: Disseminated intravascular coagulation
DS: Differentiation syndrome
ED: Early death
