# Supplementary material for: Acute promyelocytic leukemia in children cancer hospital Egypt
Source: Discov Oncol. 2024 Jun 11;15:223. doi: 10.1007/s12672-024-01037-6 (PMC11166612; doi:10.1007/s12672-024-01037-6)
Supplement: Supplementary file 1 — Additional file 1 (DOCX 216 KB) [file 12672_2024_1037_MOESM1_ESM.docx]

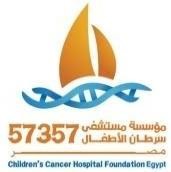


**ROADMAP OF Acute Promyelocytic Leukemia Children's Cancer Hospital Egypt (57357) Adapted from COG AAML0631**

**APL diagnosis**


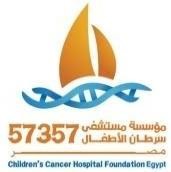
**ROADMAP OF Acute Promyelocytic Leukemia Children's Cancer Hospital Egypt (57357) Adapted from COG AAML0631**


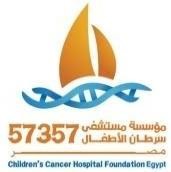
**ROADMAP OF Acute Promyelocytic Leukemia Children's Cancer Hospital Egypt (57357) Adapted from COG AAML0631**

**Maintenance- 9 cycles**

**Each cycle is 12 weeks- a total of 108 weeks**

- **ITH (ARA-C) CYCLE ONE ONLY**
- **ATRA: 25 mg/m^2^ /day, PO divided BID, 2 weeks daily, administration with food, [days 1-14] (Q 3 months).**
- **Mercaptopurine (6MP): 50mg/m^2^/day, PO, once a day**

**Administration on an empty stomach; at least 1 hour before or 2 hours after the evening meal.**

**Avoid administration with milk or citrus products.**

- **Methotrexate (MTX): 25 mg/m^2^/dose, IM, once weekly.**

**BMA for RQ-PCR analysis should be done at the end of each cycle of maintenance (Q 3 months) for all patients.**

**Supplementary Figure 1: Roadmap of acute promyelocytic leukemia, Children Cancer Hospital Egypt (57357 hospital), adopted from COG AAML0631**

**Supplementary Figure 1: ROADMAP OF Acute Promyelocytic Leukemia Children's Cancer Hospital Egypt (57357) Adapted from COG AAML0631**


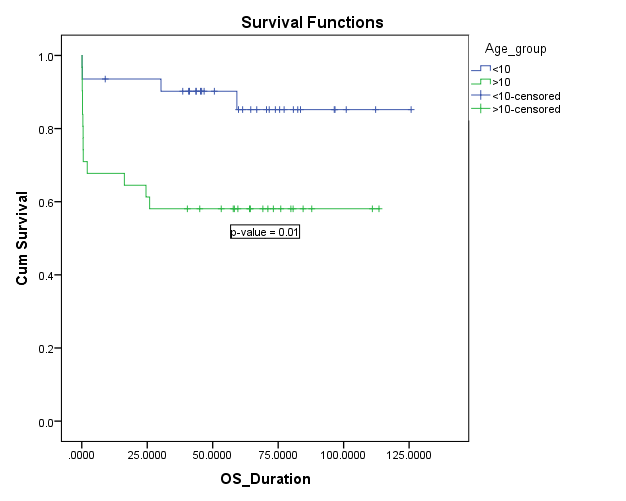


**Supplementary Figure 2: 5y overall survival according to age above and below 10 years old, which were 58.1%, and 87.1% respectively with a P-value of 0.01.**


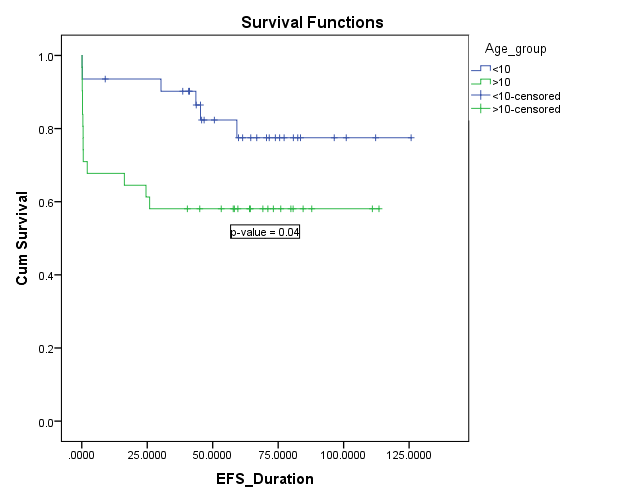


**Supplementary Figure 3: 5y Event Free Survival according to age above and below 10 years old, which were 58.1% and 80.6% respectively with a P-value 0.04.**


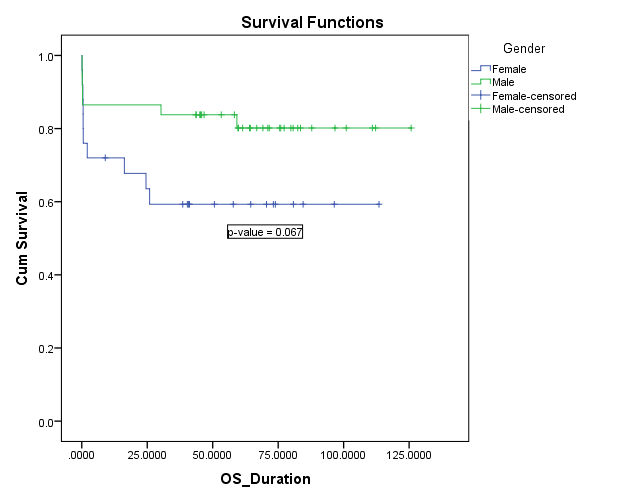


**Supplementary Figure 4: 5y overall survival according to gender (male or female) which were 81.1%, and 60.0% respectively with a P-value of 0.067.**


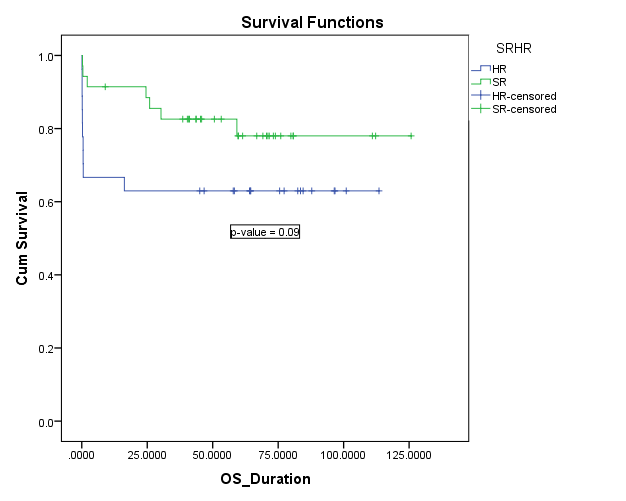


**Supplementary Figure 5: 5y overall survival according to patient’s risk (high and standard risk) which were 63.0% and 80.0% respectively with a P-value of 0.09.**


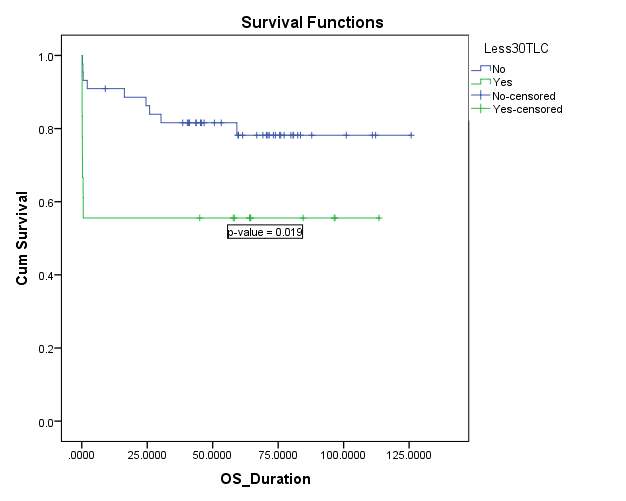


**Supplementary Figure 6: 5y overall survival according to initial TLC (above or below 30 10^3^/mm^3^) which were 55.6% and 79.5% respectively with a P- value of 0.019.**


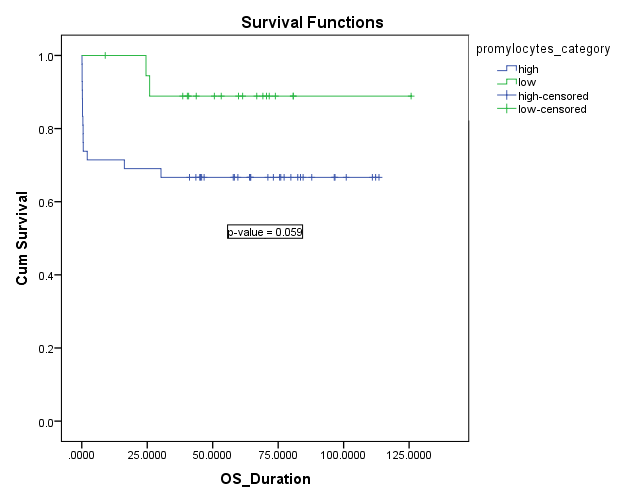


**Supplementary Figure 7: 5y overall survival according to initial promyelocytic count (above or below 10 10^3^/mm^3^) which were 66.7% and 89.5%respectively with P- value 0.059.**


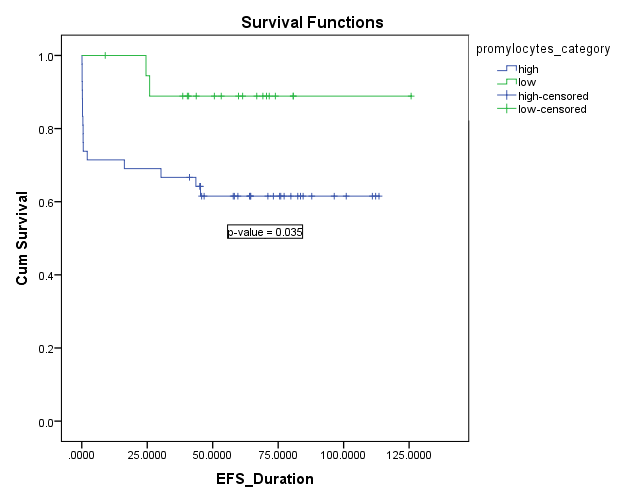


**Supplementary Figure 8: 5y Event Free Survival according to initial promyelocytic count (above or below 10 10^3^/mm^3^) which were 61.9% and 89.5% respectively with P- value 0.035.**


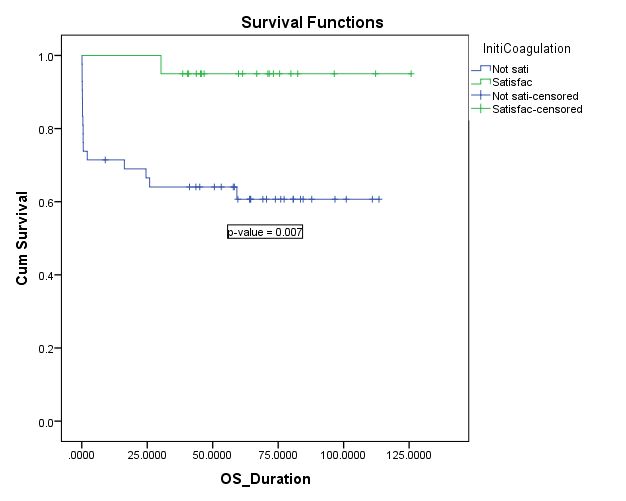


**Supplementary Figure 9: 5y overall survival according to initial coagulation profile (satisfactory and unsatisfactory) which were 95.0% and 61.9% respectively with a P-value of 0.007.**


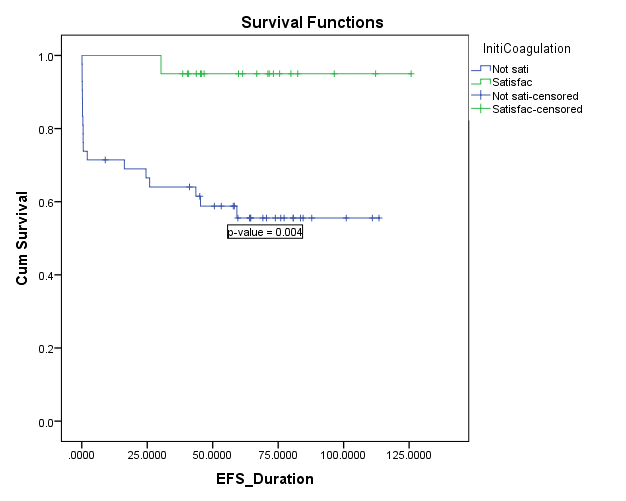


**Supplementary Figure 10: 5y Event Free Survival according to initial coagulation profile (satisfactory and unsatisfactory) which were 95.0% and 57.1% respectively with P- value of 0.004.**


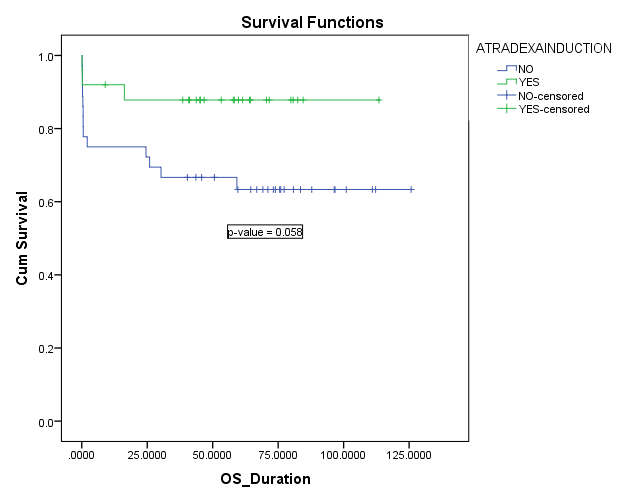


**Supplementary Figure 11: 5y overall survival according to giving steroid with ATRA or not which were, 88.0%, and 63.9% respectively with a P-value of 0.058.**


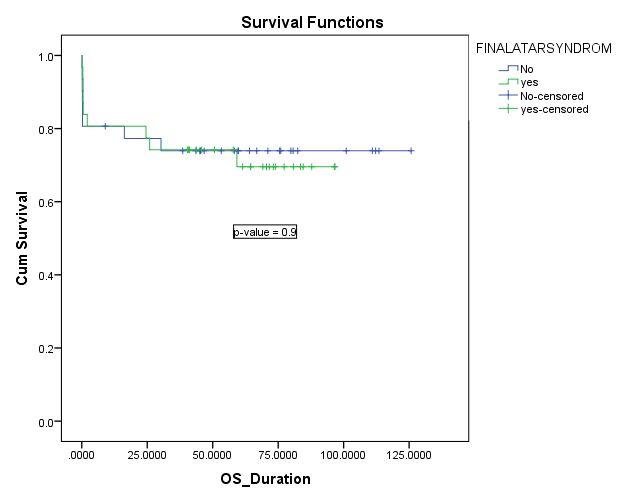


**Supplementary Figure 12: 5y overall survival according to the presence of differentiation syndrome or not which were 71.0% and 74.2% respectively with a P- value of 0.9.**


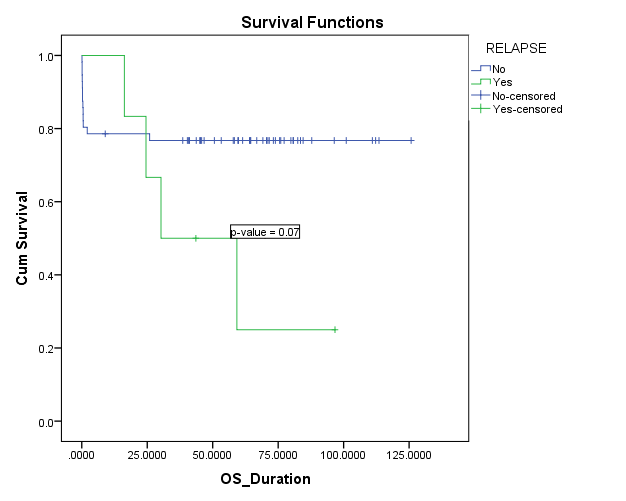


**Supplementary Figure 13: 5y overall survival according to the occurrence of relapse or not which were 33.3% and 76.8% respectively with P- value of 0.07.**


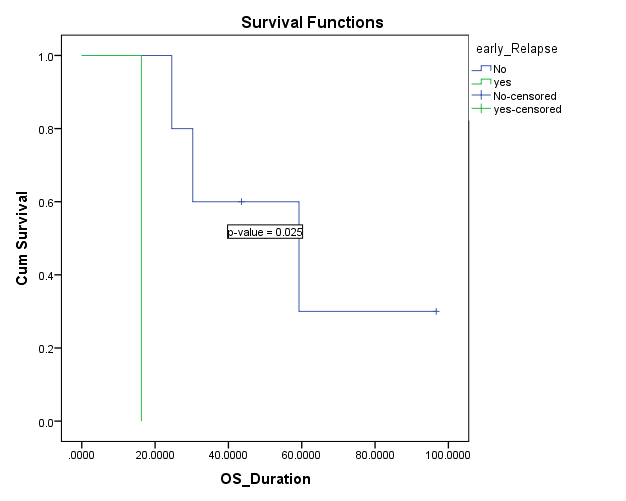


**Supplementary Figure 14: 5y overall survival according to time of relapse (early or late) which were 0.0 % and 33 % respectively with a P- value of 0.025.**

**Supplementary Table 1: statistical analysis of the age of the patients, initial TLC, blast cells, and promyelocytes.**

| **Statistics** | | | | | | |
| --- | --- | --- | --- | --- | --- | --- |
|  | | Age | Initial TLC Count (10^3^/mm^3^) | Initial Platelets Count (10^3^/mm^3^) | Peripheral Blasts% | Peripheral Promyelocytes % |
| N | Valid | 62 | 62 | 62 | 62 | 61 |
|  | Missing | 0 | 0 | 0 | 0 | 1 |
| Mean | | 9.195161 | 38.116129 | 27.02 | 18.61 | 34.89 |
| Median | | 9.950000 | 7.250000 | 19.50 | 5.00 | 28.00 |
| Range | | 16.4000 | 271.2000 | 189 | 447 | 95 |
| Minimum | | 1.4000 | 1.0000 | 5 | 0 | 0 |
| Maximum | | 17.8000 | 272.2000 | 194 | 447 | 95 |

**Supplementary Table 2: Chemotherapy cycles**

|  | Patient’s count |  |
| --- | --- | --- |
| INDUCTION 1 | 61 |  |
| ECHO | 51 satisfactory | 10 NA |
| ATRA INDUCED | 58 |  |
| differentiation syndrome (yes/no) | 27(YES) | 31(NO) |
|  |  |  |
|  | **Patient’s count** |  |
| CONSOLIDATION 1 | 50 |  |
| ECHO | 45 satisfactory | 1 unsatisfactory |
| ATRA INDUCED | 49 |  |
| differentiation syndrome | 7(YES) | 42 (NO) |
|  |  |  |
|  | **Patient’s count** |  |
| CONSOLIDATION2 | 50 |  |
| ECHO | 40 satisfactory | 1 unsatisfactory |
| ATRA INDUCED | 50 |  |
| differentiation syndrome | 7 (YES) | 43(NO) |
| MRD by PCR(positive/negative) | 38 negative | 5 Positive |
|  |  |  |
|  | **Patient’s count** |  |
| COSOLIDATION3 | 19 |  |
| ATRA INDUCED | 19 |  |
| differentiation syndrome | 2(YES) | 17(NO) |
| MRD by PCR | 8 negative | 2 Positive |
| Column1 | **Patient’s count** |  |
| MAINTENANCE | 49 |  |
| ATRA INDUCED | 49 | 44 full dose |
| differentiation syndrome | 3 (YES) | 46(NO) |
| Patients alive in complete remission | 45 | 5 years overall survival 72.5%, and with 5 years Event Free Survival 69.4%. |

| **Supplementary Table 3: Molecular MRD post-CONS II correlated to deaths** | | | | |
| --- | --- | --- | --- | --- |
| MDR PCR post CONSII | Total N | N of Events (deaths) | P-VALUE (OS) | |
|  |  |  |  |  |
| Negative | 33 | 4 | 0.38 |  |
| Positive | 5 | 0 |  |  |
| Overall | 38 | 4 |  |  |

| **Supplementary Table 4: Molecular MRD post-CONS II correlated to relapse** | | | | |
| --- | --- | --- | --- | --- |
| MDR PCR post CONSII | | RELAPSE | | P-VALUE |
|  |  | No | Yes |  |
| MDR PCR post CONSII | Negative | 33 | 5 |  |
|  | Positive | 5 | 0 | 0.47 |
|  | |  |  |  |
